# Functional performance assessment scale for children and adolescents with cancer: cross-cultural study

**DOI:** 10.1590/0034-7167-2023-0331

**Published:** 2024-06-14

**Authors:** Sandra Alves do Carmo, Isabel Cristina dos Santos Oliveira, Soraya Bactuli Cardoso, Jakcilane Rosendo de Gois, Cícero Ivan Alcantara Costa, Roberta Dantas Breia de Noronha, Tania Vignuda de Souza, Sabrina Ayd Pereira José

**Affiliations:** I Secretaria Municipal de Saúde. Duque de Caxias, Rio de Janeiro, Brazil; II Universidade Federal do Rio de Janeiro. Rio de Janeiro, Rio de Janeiro, Brazil; III Instituto Fernandes Figueira. Rio de Janeiro, Rio de Janeiro, Brazil; IV Instituto Nacional de Câncer José Alencar Gomes da Silva. Rio de Janeiro, Rio de Janeiro, Brazil; V Universidade Federal do Rio de Janeiro. Macaé, Rio de Janeiro, Brazil

**Keywords:** Cross-Cultural Comparison, Nursing Methodology Research, Physical Functional Performance, Pediatric Nursing, Oncology, Comparación Transcultural, Investigación Metodológica en Enfermería, Rendimiento Físico Funcional, Enfermería Pediátrica, Oncología

## Abstract

**Objectives:**

to describe the methodological process of cross-cultural adaptation of the PlayPerformance Scale for Children to Brazilian Portuguese.

**Methods:**

methodological study of translation and cross-cultural adaptation in six stages: translation, synthesis of translations, back-translation, evaluation by a committee of judges, evaluation by expert nurses, and pretest. The agreement and representativeness of the items were assessed using the content validity index. A minimum value of 80% agreement was considered.

**Results:**

all stages of the translation and cross-cultural adaptation process were satisfactory. In the evaluation performed by the committee of judges, all items obtained agreement above 80%. Fifteen pediatric nurses conducted the content validation, suggesting necessary modifications for understanding and application. Thirty children and adolescents with cancer were assessed with the scale for the pre-test.

**Conclusions:**

the scale was cross-culturally adapted to Brazilian Portuguese. The need for psychometric testing in a consistent sample is emphasized.

## RESUMEN

**Objetivos:**

describir el proceso metodológico de adaptación transcultural de la Escala de Rendimiento de Juego para Niños al portugués brasileño.

**Métodos:**

estudio metodológico de traducción y adaptación transcultural en seis etapas: traducción, síntesis de traducciones, retrotraducción, evaluación por un comité de jueces, evaluación por enfermeros especialistas y pretest. La concordancia y representatividad de los ítems se evaluaron mediante el índice de validez de contenido. Se consideró un valor mínimo del 80% de concordancia.

**Resultados:**

todas las etapas del proceso de traducción y adaptación transcultural fueron satisfactorias. En la evaluación realizada por el comité de jueces, todos los ítems obtuvieron una concordancia superior al 80%. Quince enfermeros pediatras realizaron la validación de contenido, sugiriendo modificaciones necesarias para la comprensión y aplicación. Treinta niños y adolescentes con cáncer fueron evaluados con la escala para el pretest.

**Conclusiones:**

la escala fue adaptada transculturalmente al portugués brasileño. Se destaca la necesidad de realizar pruebas psicométricas en una muestra consistente.

## INTRODUCTION

Pediatric and adolescent cancer corresponds to a group of various diseases characterized by the uncontrolled proliferation of abnormal cells, which can occur anywhere in the body, affecting children and adolescents aged 0 to 19 years. Unlike adult cancer, pediatric and adolescent cancer is predominantly embryonic in nature and generally affects the cells of the blood system and supporting tissues^(1)^. The most common tumors in childhood and adolescence are leukemias (affecting white blood cells) at 28%, central nervous system tumors at 26%, and lymphomas (lymphatic system) at 8%^(1)^.

The number of new cases of pediatric and adolescent cancer in Brazil, for each year of the triennium from 2023 to 2025, is 7,930 cases, corresponding to an estimated risk of 134.81 per million children and adolescents. An estimated 4,230 new cases are expected in males and 3,700 in females^(2)^. Over the past four decades, there has been significant progress in the treatment of cancer in childhood and adolescence. Today, around 80% of children and adolescents with cancer can be cured if diagnosed early and treated at specialized centers. However, in Brazil, pediatric and adolescent cancer remains the leading cause of death (8% of the total) from disease among children and adolescents aged 1 to 19 years^(1)^.

It is worth noting that the treatment of pediatric and adolescent cancer is considered complex and specialized, often requiring combined therapies (surgery, chemotherapy, and radiotherapy) aimed at increasing long-term survival. Therefore, it is essential for the healthcare team to focus on the quality of life during treatment due to the significant debilitation it can cause in children and adolescents, with the integration of palliative care being fundamental^(3)^.

The World Health Organization (WHO) proposes to integrate palliative care with curative care at the time of diagnosis and continue during and after curative treatment until death, thereby promoting quality of life for children and adolescents with cancer/ chronic disabling diseases. This translates into living life during aggressive curative treatment with quality of life and adequate control of biopsychosocial and spiritual symptoms^(4)^.

With the increasing number of cancer survivors, there is a great demand for specialized and qualified care for children and adolescents, highlighting the importance of specific assessment instruments for this population to help the healthcare team balance treatment maintenance with social and educational activities. In this sense, there is a need for further studies on the evaluation of the functionality of children and adolescents with cancer, aiming to promote quality of life.

Aware of the availability of instruments that evaluate quality of life already adapted to the Brazilian context, there has been a recognized need for cross-cultural adaptation of tools that reliably assess the functional capacity of children and adolescents. This adaptation aims to encourage activities and play conducive to functional performance, thereby fostering greater independence and autonomy to attain a satisfactory quality of life. Additionally, it underscores the importance of assessing children and adolescents with instruments tailored to their age and the Brazilian context, ensuring precise evaluation.

Hence, it is notable that the Play-Performance Scale for Children (PPSC), also referred to as the Lansky Scale, delineates the activities and play suitable for children and adolescents according to their age group. Its objective is to provide an overview of the current clinical status of the child and adolescent, grading their ability to perform activities as the disease progresses, on a scale from 100 to zero^(3)^. Developed in 1985 and validated in 1987 in the United States by Lansky and his research team, it draws on the growth and development theory outlined by Arnold Gesell, as well as the Karnofsky Performance Status scale developed for adults^(3,5)^. It is essential to highlight that functionality encompasses all bodily functions, activities, and participation^(6)^.

Healthcare professionals can utilize the PPSC scale to evaluate the effectiveness and impacts of cancer treatment, disease advancement, prognosis, and quality of life. This assessment of functional capacity acknowledges its decline, which confines children and adolescents with cancer to their beds. It is documented that children undergoing chemotherapy for cancer exhibit reduced functionality, characterized by diminished mobility, self-care, and social interaction, likely attributable to treatment side effects or the disease itself, including pain and fatigue. Consequently, their engagement in activities decreases, they spend more time in bed, and school attendance diminishes^(7)^.

Given the above, it is imperative to possess assessment tools for children and adolescents with cancer to gauge functionality and encourage the development of recreational, social, educational activities, and daily living tasks, tailored to their age, growth, and development, while monitoring disease progression. Considering the absence of a scale to assess the functional status of children and adolescents with cancer in Brazil, the translation, cross-cultural adaptation, and validation of the PPSC into Brazilian Portuguese become pertinent.

## OBJECTIVES

To describe the methodological process of cross-cultural adaptation of the Play-Performance Scale for Children (PPSC) into Brazilian Portuguese.

## METHODS

### Ethical considerations

The translation, cross-cultural adaptation of the PPSC into Brazilian Portuguese, and validation of the scale were authorized by the copyright holder of the original scale in 2018 via email. The research project was submitted to and approved by the Research Ethics Committee of the proposing and participating institutions. All study participants received the Informed Consent Form (ICF), while children (of school age) and adolescents received the Informed Assent Form (IAF), according to the institutional model. It is important to note that the study adhered to research standards involving human subjects as advocated by Resolution No. 466/12 of the National Health Council.

### Study design, duration, and location

This study involves the methodological translation and cross-cultural adaptation of the Play-Performance Scale for Children (PPSC) into Portuguese within the Brazilian context, following the recommendations outlined in the Guidelines for the Process of Cross-Cultural Adaptation of Self-Report Measures^(8)^. The study encompasses several stages: initial scale translation, synthesis of translations, back-translation, evaluation of equivalences by a committee of experts, and tests for semantic and content validity. These stages were conducted between January 2020 and March 2021. The pretest occurred from May to September 2021 in the pediatric oncology and pediatric hematology units of a leading Oncology institute in the municipality of Rio de Janeiro, Brazil.

### Population; Inclusion and Exclusion Criteria

Each stage of the study had distinct populations, inclusion, and exclusion criteria. In the stages of initial scale translation and synthesis of translations, the inclusion criteria were established as follows: agreeing to participate in the study by signing the FICF, being bilingual with proficiency in English, being over 18 years old, being a native Brazilian, and at least one translator being a healthcare professional^(8)^.

In the back-translation stage, the participation of two translators who met the following inclusion criteria was established: being American since it is a scale constructed for application in North American individuals, having lived in Brazil for at least one year, being bilingual (English and Portuguese) and over 18 years old, and agreeing to participate in the research by signing the FICF; and as exclusion criteria: having a background in healthcare and being familiar with the PPSC scale^(8)^. In the stage of scale evaluation by the committee of experts, judges who met the criteria for judge selection^(9)^ presented in Chart 1 and participants from the initial translation and synthesis stages were included.

**Chart 1:** Criteria for Judge Selection, Rio de Janeiro, Rio de Janeiro, Brazil, 2021

Criteria proposed by Fehring^(9)^:	Adapted criteria for the research:
Hold a master’s degree in nursing.	Hold a doctoral degree in nursing.
Hold a master’s degree in nursing with a dissertation in the area of diagnostic merit.	Hold a doctoral degree in nursing with a focus on pediatrics.
Have published research on diagnosis or relevant content.	Have published research on pediatrics.
Have published an article on diagnosis in an indexed journal.	Have published articles on pediatrics and methodological studies.
Have recent clinical training of at least one year in the addressed topic.	Have at least five years of clinical or teaching experience in pediatrics.
Have training in a clinical area relevant to the diagnosis of interest.	Self-report proficiency in both languages.

*Source: Adapted from Fehring^(9)^.*

During the content validation stage, nurses who met the following inclusion criteria participated in the study: having administered the PPSC scale to children and adolescents (4 points); possessing a minimum of two years’ experience in pediatric oncology (4 points); holding a doctoral degree in nursing with a pediatric focus (4 points); possessing a master’s degree in nursing with a pediatric focus (3 points); specializing in pediatric nursing (2 points); and having at least two years of experience in pediatrics (2 points). Scoring less than 4 points on the inclusion criteria served as an exclusion criterion.

In the pre-test phase, four nurses and thirty children and adolescents took part. Inclusion criteria for nurses included being registered nurses specializing in pediatrics, working in the pediatric oncology and pediatric hematology department of the Institute where data collection occurred, having available time for data collection and training, being over 18 years old, and consenting to participate in the study as data collectors. Exclusion criteria encompassed failure to complete training and being transferred from the data collection department during the research period.

For children and adolescents, inclusion criteria comprised being diagnosed with cancer, aged between 01 and 16 years, hospitalized in the pediatric oncology or pediatric hematology wards, and expressing a desire to participate in the research with their caregiver’s presence. Exclusion criteria included lacking a caregiver and/or the caregiver’s refusal to allow the child or adolescent to participate. It’s notable that the age range respected the scale’s development^(3,5)^, and the number of participants adhered to the study’s methodological guidelines^(8)^.

### Study Protocol

The method used for translation and cross-cultural adaptation followed the six stages proposed in the Guidelines for the Process of Cross-Cultural Adaptation of Self-Report Measures^(8)^. After obtaining authorization from the copyright holder of the PPSC, Stage I began with the participation of two native Brazilian translators, one English teacher, and one nurse, all fluent in the English language, resulting in two independent versions in Portuguese. In Stage II, the two translators from the first stage, along with another translator (English teacher), synthesized the translations from the two versions developed in the first stage, producing a consensus version in the Portuguese language, referred to as Version (S12)^(8)^.

In Stage III, two North American translators, residing in Brazil for more than four years, both English and Portuguese teachers, conducted back-translation (BT), independently translating Version S12 into English, resulting in versions BT1 and BT2. It is noteworthy that these two translators had no background in healthcare and were unfamiliar with the scale^(8)^.

For Stage IV, Version S12 was evaluated for semantic, idiomatic, cultural, and conceptual equivalence with the original English scale. To achieve this, the original scale versions, S12, BT1, and BT2, were sent to a committee of judges comprising six professionals: three bilingual doctoral nurse educators meeting the criteria described in Table 1 presented previously, and the translators who participated in the first and second stages of the translation process. The judges reached a consensus, resulting in the adapted scale (Version 1) after a round of independent scale evaluation regarding semantic, idiomatic, cultural, and conceptual equivalence^(8)^.

The judges assessed the equivalences using a questionnaire sent via email, consisting of the title and all items of the scale, assigning values to them regarding semantic, idiomatic, cultural, and conceptual equivalences, responding on a five-point Likert scale, with 1 for strongly disagree, 2 for disagree, 3 for undecided, 4 for agree, and 5 for strongly agree^(8)^. Subsequently, Version 1 of the scale was evaluated by fifteen pediatric nurses for content validation. Each nurse was asked to respond to an online questionnaire comprising four parts: personal data (information such as age and gender), academic background (information about postgraduate studies), professional activity (information about workplace and years of experience), and assessment of the PPSC scale (evaluation of clarity, coherence, and relevance of the scores). The questions were multiple-choice, with some open-ended questions to justify the choice, and the last part, scale evaluation, was in Likert format with five points, with 1 (strongly disagree), 2 (disagree), 3 (undecided), 4 (agree), and 5 (strongly agree). Following content validation, Version 2 emerged.

For Stage V, the pre-test, an online training session was conducted with four pediatric nurses, working in the data collection sector, to address any potential queries and evaluate the scale for clarity. Thus, a pilot test was conducted with eight children/adolescents using the adapted version (Version 2). The training was conducted virtually on a free online platform, lasting an average of two hours. A presentation and a script were developed with explanations about the data collection form items to ensure that all applicators had the same content and instructions.

The four pediatric nurse applicators were instructed to assess the children and adolescents by observing the activities and play they engaged in during hospitalization and, if necessary, supplement with an interview asking about the activities and play they could perform at the time of assessment, marking the corresponding score on the scale. Chart 2 presents the assessment script for children and adolescents using the PPSC scale discussed during the training with the pediatric nurse applicators.

**Chart 2:** Assessment Script for Children and Adolescents with the Play-Performance Scale for Children

- Check if the child and/or adolescent meets the inclusion and exclusion criteria as well as their family/caregiver; - Provide the Informed Consent Form (FICF)* and the Informed Assent Form (IAF)† to the caregiver and the child or adolescent respectively; - Explain the research to the child or adolescent and their caregiver; - Request the caregiver and the child or adolescent respectively to sign the FICF* and the IAF†; - Explain the PPSC‡ scale and its application to the caregiver; - Assess the child or adolescent by observing and, if necessary, asking about the activities they can perform at the time of assessment; - Record the start and end dates of the assessment moment and the application of the PPSC‡ scale.

*ICF - Informed Consent Form; †IAF - Informed Assent Form; ‡PPSC - Play-Performance Scale for Children.

After the pilot test, a revision of the scale in Portuguese was conducted by a linguistics professional, resulting in the Final Version (FV) used in evaluating the children and adolescents who participated in the study during the pretest. This final version was presented to the copyright holder of the scale and approved for academic use, marking the completion of Stage VI.

### Data Analysis and Statistics

In the data analysis, semantic and content validations were preceded by the Content Validity Index (CVI), which measures the proportion of agreement among judges, indicating the adequacy of the instrument concerning the study’s content. The score was calculated by summing the responses indicating “agree” and “strongly agree,” then dividing by the total number of responses^(10)^. A minimum value of 0.80 was employed for validation^(11)^. To assess the scale’s reliability and interrater consistency, Cronbach’s alpha coefficient was used, with a value greater than 0.80 indicating nearly perfect reliability^(12)^.

## RESULTS

During the initial translation and synthesis of translations, discrepancies arose between versions T1 and T2 regarding the expression “up and around,” with Translators 1 and 2 opting for the translation “awake and active.” For the terms “*veste-se*” and “*arruma sozinho*,” Translator 3 was included to impartially resolve the discrepancies, leading to the choice of “*Arruma sozinho*.”

Throughout the translation of the PPSC scale, questions arose regarding the use of the terms “*cama*” or “*leito*.” Translators 1, 2, and 3 concluded that both terms would be suitable, with “*leito*” being applicable for scale administration in a hospital setting. However, they emphasized that for administration by family members, the ideal term would be “*cama*” as it was considered more understandable. After these modifications, the translated scale version S12 emerged with 100% agreement among the translators.

In Stage III, the back-translations RT1 and RT2 were compared with the original English version (VO), resulting in 58.3% of identical translations, with other cases identifying different words but synonyms, indicating that the back-translations corresponded to the VO.

Regarding Stage IV, the committee of judges analyzed the translations, back-translations, and compared them with the original version to evaluate semantic, idiomatic, cultural, and conceptual equivalences, achieving a satisfactory total Content Validity Index (CVI): semantic equivalence (CVI=97.2), idiomatic (CVI=94.4), cultural (CVI=94.4), and conceptual (CVI=97.2).

However, two judges requested corrections in item 50 of the PPSC scale, changing “*Se arruma sozinho, mas fica prostrado na maior parte do dia... mais tranquilas*” t o “ *Se veste, mas fica deitado a maior parte do dia... mais tranquilas.*” Another adjustment requested was in the title “*Escala de desempenho do brincar para crianças*,” changed to “*Escala de desempenho de crianças no brincar/ brincadeiras*.” These adjustments were made in agreement with Translators 1, 2, and 3, resulting in the adapted version (Version 1).

Regarding reliability, the assessment among judges showed a coefficient of 0.908, considered nearly perfect, concluding semantic validation.

Before commencing Stage V of the cross-cultural adaptation process (pretest), Version 1 of the scale was evaluated by fifteen pediatric nurses for clarity, coherence, and relevance of each score contained in the scale for content validation, to assess comprehension and the need for adjustments. Overall, the evaluation of the PPSC by pediatric nurses achieved satisfactory agreements, with values exceeding the minimum stipulated of 0.8.

**Chart 3:** Original English Scale and Final Version Adapted for Brazilian Portuguese of the Play-Performance Scale for Children, Rio de Janeiro, Rio de Janeiro, Brazil, 2021

VO^*^		VF^†^
Título	Play-Performance Scale for Children	*Escala de desempenho de crianças no brincar/brincadeiras*
100	Fully active, normal	*Totalmente ativo ou normal*
90	Minor restrictions in physically strenuous activity	*Poucas restrições em atividade fisicamente exaustiva*
80	Active, but tires more quickly	*Ativo, porém, cansa-se mais rapidamente*
70	Both greater restriction of, and less time spent in, active Play	*Maior restrição e menos tempo gasto em brincadeiras ativas*
60	Up and around, but minimal active play; Keeps busy with quieter activities	*Acorda com disposição, mas realiza poucas brincadeiras ativas, mantém-se, minimamente, ocupado com brincadeiras ativas*
50	Gets dressed, but lies around much of the day; no active play; able to participate in all quiet play and activities	*Veste-se, mas fica deitado a maior parte do dia; sem participar de brincadeiras ativas. Porém, é capaz de participar de todas as brincadeiras e atividades tranquilas*
40	Mostly in bed; participates in quiet activities	*Permanece a maior parte do tempo na cama; participa de atividades tranquilas*
30	In bed; Needs assistance even for quiet play	*Na cama; necessita de assistência, mesmo para brincadeiras tranquilas*
20	Often sleeping; Play entirely limited to very passive activities	*Dorme frequentemente; brincadeiras inteiramente limitadas às atividades muito passivas*
10	No play; does not get out of bed	*Não brinca e não sai da cama*
0	Unresponsive	*Sem respost*a

*VO – Original Version; †VF – Final Version.

It is worth noting that reliability among raters was verified using Cronbach’s alpha test, obtaining 0.885, considered with nearly perfect consistency, indicating very high internal consistency. Thus, Version 2 of the PPSC scale was reached, leading to the final version (FV) after Portuguese language revision.

Regarding the characterization of children and adolescents evaluated with the PPSC scale, the most prevalent age group was 11 to 16 years old (n=11; 36.7%), followed by the age group of 2 to 5 years old (n=10; 33.3%), with a mean of 8.67 and a standard deviation of 5.261. Regarding gender, males accounted for 50.85% of the sample. The diagnosis of central nervous system tumors was the most prevalent (n=8; 26.7%). Concerning the treatment of children and adolescents, 24 (80%) recently commenced treatment, within the period of one month to one year; the majority are undergoing curative treatment (n=24; 80%), with chemotherapy being the most prevalent current treatment (n=15; 50%).

Regarding the application of the PPSC scale, the average time taken by the pediatric nurse was five minutes. However, they reported difficulties in observing activities during hospitalization, as children and adolescents engage in more passive activities and games such as using cell phones, tablets, and watching television, even with preserved functionality. Therefore, there was a need to complement the evaluation with an interview to inquire about the activities performed.

## DISCUSSION

The concern for the quality of nursing care, along with the use of appropriate and reliable assessment tools, has led to an increase in the development of methodological studies on the process of cross-cultural adaptation and validation. Although there is no standard gold methodological reference, a group of researchers^(8)^ has described stages and criteria that are followed by most researchers^(13)^.

However, some researchers^(14,15,16)^ have used methodological protocols from authors with a universalist approach^(17)^. Specifically in the pediatric area, the method of the Disabkids Group^(18)^, a European group that describes guidelines for adapting and validating instruments for children and adolescents, including semantic validation in the cross-cultural adaptation process in addition to translation, synthesis, equivalence assessment, and pretest stages. They exclude back-translation because they consider it costly^(15,16)^.

Some difficulties encountered by pediatric nurses in assessing activities and games were associated with the limited number of games and activities performed during the hospitalization period. This reduction in play usually occurs out of fear that playing may interfere with treatment or cause pain, as well as due to the healthcare professional’s overload, leaving it as a secondary concern. However, there are some play possibilities that the professional, if qualified, can suggest for pediatric clients^(19)^.

Activities and games that can be offered include conversations, storytelling, and games that can be played among the children and adolescents themselves. It is important to emphasize the importance of play for child development, given the successive prolonged hospitalizations they undergo throughout treatment^(19)^.

Other difficulties faced by pediatric nurse assessors include the need to know examples of active and passive activities or games, and the constant use of technology and electronic devices during hospitalization. It is worth noting that the nurse, as an educator, can adopt a motivating attitude towards children, adolescents, and their families to use recreational spaces such as playrooms, gardens, and courtyards^(19,20)^.

With the increasing use of technology, nurses need to delve into the topic of games and activities to update their knowledge and encourage the use of electronic games that benefit children and adolescents during hospitalization. This promotes a healthy lifestyle by associating the desire for play with the need for physical activity, which are important for maintaining good functionality^(21)^.

In the context examined, play emerges as an effective strategy to help children and adolescents cope with the enforced idleness resulting from the limited availability of activities in the hospital environment and the constant need to stay connected to medication infusion pumps, which confine them to their beds, thereby reducing functionality^(20)^.

Regarding the PPSC scale itself, doubts have arisen concerning the zero (0) score: “No response.” Understanding that functionality is a term that encompasses all bodily functions, activities, and participation^(6)^, it can be inferred that children and adolescents who, even after stimulation for any activity, play, or participation, do not produce a response should be evaluated with a score of zero (0) when in a coma or deceased state.

It is worth noting that the proposed development of the scale is related to the healthcare model where palliative care is integrated and within the cultural context of the original scale, namely American. Therefore, it is applicable to healthcare units with integrated palliative care^(3)^.

It is emphasized that assessments with the PPSC scale can be conducted whenever the healthcare professional deems it necessary, as functionality, i.e., the scale score, may vary according to the symptoms, treatment, and length of hospitalization of children and adolescents. However, further studies are suggested to evaluate the psychometric properties of the PPSC^(3,5,22)^.

In this study, chemotherapy was the most common treatment choice. Therefore, it is important to highlight that previous psychometric tests conducted on the PPSC found that cancer control treatments, especially chemotherapy, negatively influenced the functionality of children and adolescents with cancer^(22)^. Thus, the need to apply the scale in a consistent sample for further psychometric testing is emphasized.

### Study limitations

This study has some significant limitations. The main one is related to the fact that the scale has not yet undergone the process of cross-cultural adaptation in another country, which complicates the discussion and comparison of results with international studies. Additionally, it is worth noting that the principal researcher did not participate in data collection, limiting the conduct of further psychometric tests. Another aspect to consider is the reduction in activities for children and adolescents in the hospital environment due to restrictions on people’s movement in healthcare facilities, as a measure to control the spread of the SARS-CoV-2 virus.

### Contributions to Nursing and Public Health

This study contributes to the advancement of scientific knowledge and nursing by providing a scale in Portuguese, adapted to the Brazilian reality, which assesses functionality based on the activities and play developed by children and adolescents, with the aim of promoting their autonomy and independence during hospitalization, thereby facilitating early discharge to home. The PPSC scale, in its Portuguese version, can be reliably used in the evaluation of other scales, as it has been translated and adapted scientifically through recognized protocols widely used by the scientific community. Thus, instrument validation studies may use the adapted version for concurrent and convergent validation regarding disorders that cause a decrease in children’s and adolescents’ functionality or alteration in quality of life.

## CONCLUSIONS

The scale was translated and adapted for Brazilian Portuguese and proved to be relevant to the healthcare and nursing field by assessing the functionality of children and adolescents with cancer, facilitating the identification and stimulation of activities and play according to individual functional capacity, as well as indicating the need for further studies to assess functionality reliably using a scale tailored to the Brazilian reality.
