# Supplementary material for: Functional performance assessment scale for children and adolescents with cancer: cross-cultural study
Source: Rev Bras Enferm. 2024 Jun 14;77(2):e20230331. doi: 10.1590/0034-7167-2023-0331 (PMC11178304; doi:10.1590/0034-7167-2023-0331)
Supplement: Translation

# Translation: Escala de avaliação do desempenho funcional de crianças e adolescentes com câncer: estudo transcultural

**DOI:** 10.1590/0034-7167-2023-0331pt

**Authors:** Sandra Alves do Carmo, Isabel Cristina dos Santos Oliveira, Soraya Bactuli Cardoso, Jakcilane Rosendo de Gois, Cícero Ivan Alcantara Costa, Roberta Dantas Breia de Noronha, Tania Vignuda de Souza, Sabrina Ayd Pereira José

**Affiliations:** I Secretaria Municipal de Saúde. Duque de Caxias, Rio de Janeiro, Brasil; II Universidade Federal do Rio de Janeiro. Rio de Janeiro, Rio de Janeiro, Brasil; III Instituto Fernandes Figueira. Rio de Janeiro, Rio de Janeiro, Brasil; IV Instituto Nacional de Câncer José Alencar Gomes da Silva. Rio de Janeiro, Rio de Janeiro, Brasil; V Universidade Federal do Rio de Janeiro. Macaé, Rio de Janeiro, Brasil

**Keywords:** Comparação Transcultural, Pesquisa Metodológica em Enfermagem, Desempenho Físico Funcional, Enfermagem Pediátrica, Oncologia

## Abstract

**Objetivos:**

descrever o processo metodológico de adaptação transcultural da *Play-Performance Scale for Children* para o português brasileiro.

**Métodos:**

estudo metodológico de tradução e adaptação transcultural em seis etapas: tradução, síntese das traduções, retrotradução, avaliação por um comitê de juízes, avaliação por enfermeiros especialistas e pré-teste. A concordância e a representatividade dos itens foram avaliadas por meio do índice de validade de conteúdo. Considerou-se o valor mínimo de 80% de concordância.

**Resultados:**

todas as etapas do processo de tradução e adaptação transcultural foram satisfatórias. Na avaliação realizada pelo comitê de juízes, todos os itens obtiveram concordância superior a 80%. Quinze enfermeiros pediatras realizaram a validação de conteúdo, sugerindo modificações necessárias para o entendimento e aplicação. Trinta crianças e adolescentes com câncer foram avaliados com a escala para o pré-teste.

**Conclusões:**

a escala foi adaptada transculturalmente para o português brasileiro. Ressalta-se a necessidade da realização de testes psicométricos em uma amostra consistente.

## INTRODUÇÃO

O câncer infantojuvenil corresponde a um grupo de várias doenças que têm em comum a proliferação descontrolada de células anormais e que pode ocorrer em qualquer local do organismo, acometendo crianças e adolescentes na faixa etária de 0 a 19 anos. Diferentemente do câncer do adulto, o câncer infantojuvenil é predominantemente de natureza embrionária e, geralmente, afeta as células do sistema sanguíneo e os tecidos de sustentação^(1)^. Os tumores mais frequentes na infância e na adolescência são as leucemias (que afetam os glóbulos brancos) com 28%, os do sistema nervoso central com 26% e linfomas (sistema linfático) com 8%^(1)^.

O número de casos novos de câncer infantojuvenil no Brasil, para cada ano do triênio de 2023 a 2025, é de 7.930 casos, o que corresponde a um risco estimado de 134,81 por milhão de crianças e adolescentes. Estimam-se 4.230 casos novos no sexo masculino e 3.700 no sexo feminino^(2)^. Nas últimas quatro décadas, o progresso no tratamento do câncer na infância e na adolescência foi extremamente significativo. Hoje, em torno de 80% das crianças e adolescentes acometidos de câncer podem ser curados, se diagnosticados precocemente e tratados em centros especializados^(1)^. Porém, no Brasil, o câncer infantojuvenil ainda é a primeira causa de morte (8% do total) por doença entre crianças e adolescentes de 1 a 19 anos^(1)^.

Cabe ressaltar que o tratamento do câncer infantojuvenil é considerado complexo, especializado e, na maioria das vezes, necessita de terapias combinadas (cirurgia, quimioterapia e radioterapia) visando aumentar a sobrevida a longo prazo^(3)^. Com isso, é necessário que a equipe de saúde atente para a qualidade de vida também durante o tratamento devido à alta debilidade que pode causar na criança e no adolescente, sendo fundamental a integração da assistência paliativa^(3)^.

A proposta da Organização Mundial de Saúde (OMS) é de integrar a assistência paliativa à curativa no momento do diagnóstico e continuar durante e após o tratamento curativo até o momento da morte, e assim, promover a qualidade de vida para crianças e adolescentes com câncer/doenças crônicas incapacitantes, o que se traduz em viver o tempo de vida durante o tratamento curativo agressivo com qualidade de vida e controle adequado dos sintomas biopsicossociais e espirituais^(4)^.

Com o crescente aumento de sobreviventes do câncer, existe uma grande demanda de crianças e adolescentes com necessidade de atendimento especializado e qualificado, tornando evidente a importância de instrumentos de avaliação específicos para esta clientela que auxiliem a equipe de saúde a conciliar a manutenção do tratamento com as atividades sociais e educacionais. Nesse sentido, destaca-se a necessidade de novos estudos sobre a avaliação da funcionalidade da criança e do adolescente com câncer com vistas à promoção da qualidade de vida.

Sabendo da existência de instrumentos que avaliam a qualidade de vida já adaptados para a realidade brasileira, observou-se a necessidade de adaptação transcultural de instrumentos que avaliem a capacidade funcional de crianças e adolescentes de forma fidedigna com o intuito de estimular atividades e brincadeiras adequadas ao desempenho funcional, proporcionando maior independência e autonomia para alcançar a qualidade de vida. Ressalta-se também a necessidade de avaliar crianças e adolescentes com instrumentos adequados à idade e à realidade brasileira, visando uma avaliação precisa.

Assim, cabe destacar que a *Play-Performance Scale for Children* (PPSC), também conhecida como Escala de Lansky, é uma escala que descreve as atividades e brincadeiras que crianças e adolescentes conseguem desenvolver de acordo com sua faixa etária, com o objetivo de proporcionar uma visão geral do estado clínico atual da criança e do adolescente através da graduação em decis (de 100 a zero) das atividades que eles conseguem desenvolver com o avanço da doença^(3)^. A PPSC foi elaborada em 1985 e validada em 1987 nos Estados Unidos da América por Lansky e por seu grupo de pesquisa, com base na teoria de crescimento e desenvolvimento descrita por Arnold Gesell e na escala de avaliação da funcionalidade desenvolvida para adultos KPS (*Performance Status de Karnofsky*) ^(3,5)^. Cabe ressaltar que a funcionalidade é um termo que abrange todas as funções do corpo, atividades e participação^(6)^.

A escala PPSC pode ser aplicada por profissionais de saúde com o objetivo de avaliar a eficácia e os efeitos do tratamento do câncer, a progressão da doença, o prognóstico e a qualidade de vida, através da avaliação da capacidade funcional, por entender que os mesmos diminuem a funcionalidade, restringindo a criança e o adolescente com câncer ao leito^(3)^. Sabe-se que a criança com câncer em tratamento quimioterápico apresenta funcionalidade diminuída, sendo observada redução da mobilidade, do auto-cuidado e da interação social, possivelmente ocasionados pelos efeitos adversos do tratamento ou decorrentes da própria doença como dor e fadiga. Desta forma, observa-se menor participação da criança nas atividades, maior tempo no leito e baixa frequência em ambiente escolar^(7)^.

Diante do exposto, faz-se necessário instrumentos de avaliação das crianças e adolescentes com câncer para medir a funcionalidade e estimular o desenvolvimento das atividades lúdicas, sociais, escolares e atividades de vida diária de acordo com a idade e o crescimento e desenvolvimento; atentando para a progressão da doença. Considerando não ter disponível uma escala para avaliação funcional de crianças e adolescentes com câncer no Brasil, torna-se relevante a tradução e adaptação transcultural da PPSC para o português do Brasil, bem como a validação para a realidade brasileira.

## OBJETIVOS

Descrever o processo metodológico de adaptação transcultural da *Play-Per formance Scale for Children* (PPSC) para o por tuguês brasileiro.

## MÉTODOS

### Aspectos éticos

A tradução e a adaptação transcultural para o português brasileiro e a validação da escala PPSC foram autorizadas pelo detentor dos direitos autorais da escala original em 2018 por correio eletrônico. O projeto de pesquisa foi submetido e aprovado pelo Comitê de Ética em Pesquisa da instituição proponente e da instituição coparticipante. Todos os participantes do estudo receberam o Termo de Consentimento Livre e Esclarecido (TCLE), e as crianças (em idade escolar) e os adolescentes o Termo de Assentimento Informado (TAI), conforme o modelo institucional. Cabe destacar que o estudo obedeceu às normas de pesquisa envolvendo seres humanos preconizadas pela Resolução nº 466/12 do Conselho Nacional de Saúde.

### Desenho, período e local do estudo

Trata-se de um estudo metodológico de tradução e adaptação transcultural da escala de avaliação da funcionalidade *Play-Performance Scale for Children* (PPSC) para o português no contexto brasileiro, de acordo com as recomendações propostas pelo *Guidelines for the Process of Cross-Cultural Adaptation of Self-Report Measures* ^(8)^. O estudo compreendeu as seguintes etapas: tradução inicial da escala, síntese das traduções, retrotradução, avaliação das equivalências por um comitê de especialistas, testes para validade semântica e de conteúdo, sendo essas etapas realizadas entre janeiro de 2020 e março de 2021. Quanto ao pré-teste, este ocorreu nos meses de maio a setembro de 2021 nas unidades de oncologia pediátrica e hematologia infantil de um Instituto de referência em Oncologia no município do Rio de Janeiro, Brasil.

### População; critérios de inclusão e exclusão

Cada etapa do estudo teve população, critérios de inclusão e exclusão distintos. Nas etapas de tradução inicial da escala e síntese das traduções, estabeleceu-se como critérios de inclusão: aceitar participar do estudo preenchendo o TCLE, serem bilingues com proficiência em inglês com mais de 18 anos de idade, brasileiros natos e pelo menos um tradutor ser profissional da saúde^(8)^.

Na etapa da retrotradução, estabeleceu-se a participação de dois tradutores que atendessem aos seguintes critérios de inclusão: ser norte-americano por se tratar de uma escala construída para aplicação em pessoas norte-americanas, morarem no Brasil a pelo menos um ano, ser bilingue (inglês e português) com idade superior a 18 anos e que aceitasse participar da pesquisa assinando o TCLE; e como critérios de exclusão: ter formação na área da saúde e conhecer a escala PPSC^(8)^. Na etapa de avaliação da escala pelo comitê de experts, incluiu-se os juízes que atenderam aos critérios para seleção dos juízes^(9)^ apresentado no Quadro 1 e os participantes das etapas de tradução inicial e síntese.

**Quadro 1:** Critérios para a Seleção dos juízes, Rio de Janeiro, Rio de Janeiro, Brasil, 2021

Critérios propostos por Fehring^(9)^	Critérios adaptados para a pesquisa
Ser mestre em enfermagem	Ser doutor em enfermagem
Ser mestre em enfermagem, com dissertação na área de mérito do diagnóstico	Ser doutor em enfermagem, com doutorado na área pediátrica
Ter pesquisa publicada sobre diagnóstico ou conteúdo relevante	Ter pesquisa publicada sobre pediatria
Ter artigo publicado sobre diagnóstico, em periódico indexado	Ter artigo publicado sobre pediatria e estudos metodológicos
Ter capacitação clínica recente de no mínimo um ano, na temática abordada	Ter prática clínica ou docente de no mínimo cinco anos em pediatria
Ter capacitação em área clínica relevante ao diagnóstico de interesse	Ser bilingue autorrelatada

*Fonte: Adaptado de Fehring^(9)^.*

Na etapa de validação do conteúdo, participaram do estudo enfermeiros que atenderam aos seguintes critérios de inclusão: Ter aplicado a escala PPSC em crianças e adolescentes (4 pontos); ter no mínimo dois anos de experiência em oncologia pediátrica (4 pontos); ter doutorado em enfermagem com temática pediátrica (4 pontos); ter mestrado em enfermagem com temática pediátrica (3 pontos); ter especialização em enfermagem pediátrica (2 pontos) e ter no mínimo dois anos de experiência em pediatria (2 pontos). Como critério de exclusão, foi considerado ter pontuado menos de 4 pontos nos critérios de inclusão.

Na etapa de pré-teste, participaram quatro enfermeiros e trinta crianças e adolescentes. Os critérios de inclusão para a participação dos enfermeiros foram: ser enfermeiro graduado e com especialização em pediatria, atuar no setor de oncologia pediátrica e hematologia infantil do Instituto onde ocorreu a coleta de dados, ter disponibilidade de tempo para a coleta de dados e para realizar o treinamento, ser maior de 18 anos e aceitar participar do estudo na condição de pesquisador coletador. Como critério de exclusão, definiu-se não concluir o treinamento e ser transferido do setor de coleta de dados no período da pesquisa.

Os critérios de inclusão para as crianças e adolescentes foram: ser criança ou adolescente com diagnóstico de câncer, ter idade entre 01 e 16 anos, estar internada nas enfermarias de oncologia pediátrica ou hematologia infantil, expressar desejo de participar da pesquisa através do TAI perante seu acompanhante. Como critério de exclusão, definiu-se estar sem acompanhante e/ou o acompanhante não concordar com a participação da criança ou do adolescente. Cabe destacar que as idades foram determinadas respeitando as idades para as quais a escala foi elaborada^(3,5)^ e o número de crianças e/ou adolescentes respeitou o referencial metodológico do estudo^(8)^.

### Protocolo do estudo

O método utilizado para tradução e adaptação transcultural seguiu as seis etapas propostas no *Guidelines for the Process of Cross-Cultural Adaptation of Self-Report Measures* ^(8)^. Após a autorização do detentor dos direitos autorais da PPSC, iniciou-se a Etapa I com a participação de duas tradutoras brasileiras natas, uma professora de inglês e outra enfermeira, fluentes na língua inglesa, emergindo duas versões na língua portuguesa de forma independente. Na Etapa II, as duas tradutoras participantes da primeira etapa, mais uma tradutora (professora de inglês) realizaram a síntese das traduções a partir das duas versões elaboradas na primeira etapa, produzindo de forma consensual a versão no idioma português sendo denominada versão (S12)^(8)^.

Na Etapa III, dois tradutores norte-americanos, residentes no Brasil há mais de quatro anos, professores de inglês e português realizaram a retrotradução (RT), traduzindo a versão S12 para o inglês de forma independente, surgindo as versões RT1 e RT2. Ressalta-se que os dois tradutores não têm formação na área da saúde e desconheciam a escala^(8)^.

Para a Etapa IV, a versão S12 foi avaliada quanto a equivalência semântica, idiomática, cultural e conceitual com a escala original em inglês. Para isto, as versões da escala original, S12, RT1 e RT2 foram encaminhadas para um comitê de juízes formado por seis profissionais: três enfermeiras doutoras e orientadoras bilingues atendendo aos critérios descritos no Quadro 1 já apresentado, e os tradutores participantes das primeira e segunda etapas do processo de tradução. Os juízes chegaram a um consenso emergindo a escala adaptada (versão 1) após uma rodada de avaliação da escala de forma independente quanto a equivalência semântica, idiomática, cultural e conceitual^(8)^.

Os juízes avaliaram as equivalências através de um questionário enviado por endereço eletrônico composto por título e por todos os itens da escala atribuindo valores aos mesmos quanto a equivalências semântica, idiomática, cultural e conceitual, respondendo a uma escala tipo Likert de cinco pontos sendo 1 para discordo totalmente, 2 para discordo, 3 para indeciso, 4 para concordo e 5 para concordo totalmente^(8)^. Em seguida, a escala versão 1 foi avaliada por quinze enfermeiros pediatras para validação do conteúdo. Foi solicitado a cada enfermeiro que respondessem a um questionário on-line composto por quatro partes: dados pessoais (informações como idade e sexo), formação acadêmica (informações sobre pós-graduação), atividade profissional (informações sobre local de trabalho e tempo de experiência) e a avaliação da escala PPSC (avaliação sobre clareza, coerência e pertinência dos escores). As perguntas eram de múltipla escolha e algumas dissertativas para justificar a escolha e, a última parte, avaliação da escala, em formato tipo Likert com cinco pontos sendo 1 (discordo totalmente), 2 (discordo), 3 (indeciso), 4 (concordo) e 5 (concordo totalmente). Após a validação de conteúdo, emergiu a Versão 2.

Para a Etapa V, pré-teste, realizou-se um treinamento online com quatro enfermeiros pediatras, atuantes no setor de coleta de dados, a fim de sanar possíveis dúvidas e avaliarem a escala quanto a clareza. Assim, optou-se pelo teste piloto com oito crianças/adolescentes utilizando a versão adaptada (versão 2). O treinamento foi virtual em plataforma disponibilizada on-line e gratuita e teve durabilidade média de duas horas. Elaborou-se uma apresentação e um roteiro com explicações sobre os itens do formulário de coleta de dados para garantir que todos os aplicadores tivessem o mesmo conteúdo e as mesmas orientações.

Os quatro enfermeiros pediatras aplicadores foram orientados a avaliar as crianças e os adolescentes através da observação das atividades e brincadeiras realizadas durante a internação e, se necessário, complementar com uma entrevista perguntando sobre quais atividades e brincadeiras conseguiam realizar no momento da avaliação, marcando na escala o escore correspondente a avaliação. O Quadro 2 apresenta o roteiro de avaliação da criança e do adolescente com a escala PPSC discutido no treinamento com os enfermeiros pediatras aplicadores.

**Quadro 2:** Roteiro de avaliação da criança e do adolescente com a *Play-Performance Scale for Children*

- Checar se a criança e/ou adolescente atendem aos critérios de inclusão e exclusão bem como o seu familiar/acompanhante; - Entregar o Termo de Consentimento Livre e Esclarecido (TCLE)^*^ e o Termo de Assentimento Informado (TAI)^†^ ao familiar-acompanhante e à criança ou adolescente respectivamente; - Explicar a pesquisa à criança ou adolescente e ao seu familiar-acompanhante; - Solicitar a assinatura do TCLE^*^ e do TAI^†^ ao familiar-acompanhante e à criança ou adolescente respectivamente; - Explicar a escala PPSC^‡^ e sua aplicação ao familiar-acompanhante; - Avaliar a criança ou adolescente observando e, se necessário, perguntando sobre as atividades que consegue desenvolver no momento da avaliação; - Colocar a data de início e término do momento de avaliação e aplicação da escala PPSC^‡^.

^*^TCLE – Termo de Consentimento Livre e Esclarecido; ^†^TAI – Termo de Assentimento Informado; ^‡^PPSC – Play-Performance Scale for Children.

Por fim, após o teste piloto, foi realizada uma revisão de português da escala por uma profissional de linguística, emergindo a Versão Final (VF) aplicada na avaliação das crianças e adolescentes, participantes do estudo durante o pré-teste e apresentada ao detentor dos direitos autorais da escala, sendo aprovada para uso acadêmico, completando a Etapa VI.

### Análise dos resultados e estatística

Na análise dos dados, as validações semântica e de conteúdo foram precedidas pela realização do Índice de Validade de Conteúdo (IVC), que mede a proporção de concordância entre juízes, representando a adequação do instrumento em relação ao conteúdo do estudo. O escore foi calculado somando as respostas dos valores referentes a “concordo” e “concordo totalmente”, dividido pelo total de respostas^(10)^. Para a validação, foi empregado o valor mínimo de 0,80^(11)^. Para medir a confiabilidade da escala e entre os avaliadores (consistência interna), foi utilizado o coeficiente alfa de Cronbach, considerando um valor maior que 0,80 como quase perfeito^(12)^.

## RESULTADOS

Durante a tradução inicial e a síntese das traduções, surgiram divergências entre as versões T1 e T2 relacionadas à expressão “*up and around*”, sendo escolhida pelas Tradutoras 1 e 2 a tradução “acorda com vigor”. Para as palavras “veste-se” e “arruma sozinho”, houve a inclusão da Tradutora 3 para solucionar as divergências de forma imparcial, optando-se por “Arruma sozinho”.

No decorrer da tradução da escala PPSC, houve questionamentos quanto ao uso dos termos “cama” ou “leito”. As tradutoras 1, 2 e 3 concluíram que ambos os termos seriam adequados, podendo usar “leito” para aplicação da escala no ambiente hospitalar. Contudo, ressaltaram que, para aplicação pelo familiar, o ideal seria utilizar o termo “cama” por ser considerado mais compreensível. Após essas modificações, emergiu a escala traduzida versão S12 com concordância de 100% entre as tradutoras.

Na etapa III, as retrotraduções RT1 e RT2 foram equiparadas com a versão original (VO) em inglês, obtendo 58,3% de traduções idênticas e, nos demais casos, foram identificadas palavras diferentes, porém sinônimas, concluindo que as traduções reversas correspondiam à VO.

No que diz respeito à etapa IV, o comitê de juízes analisou as traduções, as retrotraduções e comparou com a versão original para avaliação das equivalências semântica, idiomática, cultural e conceitual, obtendo um IVC total satisfatório: equivalência semântica (IVC=97,2), idiomática (IVC=94,4), cultural (IVC=94,4) e conceitual (IVC=97,2).

Entretanto, dois juízes solicitaram correções no escore 50 da escala PPSC, alterando “Se arruma sozinho, mas fica prostrado na maior parte do dia... mais tranquilas” para “Se veste, mas fica deitado a maior parte do dia... mais tranquilas”. Outro ajuste solicitado foi no título “Escala de desempenho do brincar para crianças”, alterando-o para “Escala de desempenho de crianças no brincar/brincadeiras”. Tais ajustes foram realizados de acordo com a concordância das Tradutoras 1, 2 e 3, resultando na versão adaptada (Versão 1).

Com relação à confiabilidade, a avaliação entre os juízes apresentou um coeficiente de 0,908, considerado quase perfeito, concluindo a validação semântica.

Antes de iniciar a etapa V do processo de adaptação trans-cultural (pré-teste), a Versão 1 da escala foi avaliada por quinze enfermeiros pediatras quanto à clareza, coerência e pertinência de cada escore contido na escala para validação de conteúdo, a fim de verificar a compreensão e a necessidade de ajustes. No geral, a avaliação da PPSC pelos enfermeiros pediatras obteve concordâncias satisfatórias, alcançando valores acima do mínimo estipulado de 0,8.

Vale ressaltar que foi verificada a confiabilidade entre os avaliadores com o teste alfa de Cronbach, obtendo 0,885, considerado com uma consistência quase perfeita, evidenciando uma consistência interna muito elevada. Com isso, chegou-se à Versão 2 da escala PPSC, e posteriormente à versão final (VF) após revisão de português.

Quanto à caracterização das crianças e dos adolescentes com câncer avaliados com a escala PPSC, a faixa etária de maior prevalência foi de 11 a 16 anos (n=11; 36,7%), seguida da faixa etária de 2 a 5 anos (n=10; 33,3%), com média de 8,67 e desvio padrão de 5,261. Quanto ao sexo, o masculino correspondeu a 50,85% da amostra. O diagnóstico de tumor de sistema nervoso central foi o mais prevalente (n=8; 26,7%). A respeito do tratamento das crianças e dos adolescentes, 24 (80%) iniciaram o tratamento recentemente, no período de um mês a um ano; a maioria está fazendo tratamento curativo (n=24; 80%), sendo a quimioterapia, o tratamento atual mais prevalente (n=15; 50%).

Em relação à aplicação da escala PPSC, o tempo médio pelo enfermeiro pediatra foi de cinco minutos. Contudo, eles relataram dificuldades em observar as atividades durante a internação, visto que as crianças e os adolescentes quando estão internados, desenvolvem atividades e brincadeiras mais passivas como uso de celulares, tablets e assistem televisão, mesmo com a funcionalidade preservada. Com isso, houve a necessidade de complementar a avaliação com uma entrevista para saber sobre as atividades realizadas. Quanto à caracterização das crianças e dos adolescentes com câncer avaliados com a escala PPSC**,** a faixa etária de maior prevalência foi de 11 a 16 anos (n=11; 36,7%) seguida da faixa etária de 2 a 5 anos (n=10; 33,3%), com média de 8,67 e desvio padrão de 5,261. Quanto ao sexo, o masculino correspondeu a 50,85% da amostra. O diagnóstico de tumor de sistema nervoso central foi o mais prevalente (n=8; 26,7%). A respeito do tratamento das crianças e dos adolescentes, 24 (80%) iniciaram o tratamento recentemente, no período de um mês a um ano; a maioria está fazendo tratamento curativo (n=24; 80%), sendo a quimioterapia, o tratamento atual mais prevalente (n=15; 50%).

**Quadro 3:** Escala original em inglês e Versão Final adaptada para o português brasileiro da *Play-Performance Scale for Children*, Rio de Janeiro, Rio de Janeiro. Brasil, 2021

VO^*^		VF^†^
Título	*Play-Performance Scale for Children*	Escala de desempenho de crianças no brincar/brincadeiras
100	*Fully active, normal*	Totalmente ativo ou normal
90	*Minor restrictions in physically strenuous activity*	Poucas restrições em atividade fisicamente exaustiva
80	*Active, but tires more quickly*	Ativo, porém, cansa-se mais rapidamente
70	*Both greater restriction of, and less time spent in, active Play*	Maior restrição e menos tempo gasto em brincadeiras ativas
60	*Up and around, but minimal active play; Keeps busy with quieter activities*	Acorda com disposição, mas realiza poucas brincadeiras ativas, mantém-se, minimamente, ocupado com brincadeiras ativas
50	*Gets dressed, but lies around much of the day; no active play; able to participate in all quiet play and activities*	Veste-se, mas fica deitado a maior parte do dia; sem participar de brincadeiras ativas. Porém, é capaz de participar de todas as brincadeiras e atividades tranquilas
40	*Mostly in bed; participates in quiet activities*	Permanece a maior parte do tempo na cama; participa de atividades tranquilas
30	*In bed; Needs assistance even for quiet play*	Na cama; necessita de assistência, mesmo para brincadeiras tranquilas
20	*Often sleeping; Play entirely limited to very passive activities*	Dorme frequentemente; brincadeiras inteiramente limitadas às atividades muito passivas
10	*No play; does not get out of bed*	Não brinca e não sai da cama
0	*Unresponsive*	Sem resposta

*VO – Versão Original; ^†^VF – Versão Final.

## DISCUSSÃO

A preocupação com a qualidade da assistência de enfermagem, com uso de instrumentos avaliativos adequados e confiáveis, tem aumentado o desenvolvimento de estudos metodológicos sobre o processo de adaptação transcultural e validação. Apesar de não haver referencial metodológico padrão-ouro, um grupo de pesquisadores^(8)^ descreveu etapas e critérios que são seguidos pela maior parte dos pesquisadores^(13)^.

Contudo, alguns pesquisadores^(14,15,16)^ utilizaram em seus estudos protocolos metodológicos de autores com uma perspectiva de abordagem universalista^(17)^ e, especificamente na área pediátrica, o método do Grupo *Disabkids* ^(18)^, grupo Europeu que descreve diretrizes para serem utilizadas na adaptação e validação de instrumentos para crianças e adolescentes, incluindo a validação semântica no processo de adaptação transcultural além das etapas de tradução, síntese, avaliação das equivalências e pré-teste, excluindo a retrotradução por entenderem que seria dispendioso^(15,16)^.

Algumas dificuldades encontradas pelos enfermeiros pediatras em avaliar as atividades e brincadeiras estavam associadas a poucas brincadeiras e atividades realizadas durante o período de internação. Tal redução de brincadeiras geralmente ocorre por medo de que o brincar interfira no tratamento e ou cause dor, como também devido à sobrecarga do profissional de saúde deixando para segundo plano. Porém existem algumas possibilidades de brincadeiras que o profissional, estando habilitado, pode propor para a clientela pediátrica^(19)^.

As atividades e as brincadeiras que podem ser oferecidas estão relacionadas a conversas, a contação de histórias e as brincadeiras que podem ser realizadas entre as próprias crianças e adolescentes. Cabe ressaltar a importância do brincar para o desenvolvimento infantil visto as sucessivas internações prolongadas às quais elas estão submetidas ao longo do tratamento^(19)^.

Outras dificuldades dos enfermeiros pediatras avaliadores foram a necessidade de saber exemplos sobre atividades ou brincadeiras ativas e passivas e o constante uso de tecnologia e dispositivos eletrônicos durante a internação. Vale lembrar que o enfermeiro, por ter um papel educador, pode ter uma postura motivadora para as crianças e para os adolescentes bem como suas famílias a utilizarem os espaços recreativos como a brinquedoteca, jardins e pátios^(19,20)^.

Com o aumento do uso da tecnologia, o enfermeiro precisa se aprofundar na temática sobre brincadeiras e atividades para atualizar seus conhecimentos e encorajar o uso de jogos eletrônicos que beneficiem as crianças e os adolescentes no período de internação, promovendo o estilo de vida saudável, associando ao desejo da brincadeira a necessidade da atividade física, as quais são importantes para manterem uma boa funcionalidade^(21)^.

O brincar, no contexto estudado, configura-se como estratégia eficaz para auxiliar crianças e adolescentes no enfrentamento da ociosidade imposta pela pouca oferta de atividades no ambiente hospitalar e pela constante necessidade de manterem-se conectadas às bombas de infusão de medicamentos que as restringem no leito, diminuindo a funcionalidade^(20)^.

Quanto à escala PPSC, propriamente dita, surgiram dúvidas relacionadas ao escore zero (0): “Sem resposta”. Sabendo que a funcionalidade é um termo que abrange todas as funções do corpo, atividades e participação^(6)^, pode-se entender que as crianças e os adolescentes que, mesmo após o estímulo para alguma atividade, brincadeira ou participação, não produzam resposta, sugere-se avaliar com o escore zero (0) quando em estado de coma ou óbito.

Vale destacar que a proposta de elaboração da escala está relacionada com o modelo de assistência à saúde onde os cuidados paliativos estão inseridos e no contexto cultural da escala original, ou seja, norte-americano, portanto, em unidades de saúde com a integralização da assistência paliativa^(3)^.

Ressalta-se que as avaliações com a escala PPSC podem ser realizadas sempre que o profissional de saúde achar necessário, visto que a funcionalidade, ou seja, o escore da escala, pode variar de acordo com os sintomas, tratamento e tempo de internação das crianças e dos adolescentes. Porém, sugere-se mais estudos para avaliar as propriedades psicométricas da PPSC^(3,5,22)^.

Neste estudo, a quimioterapia foi o tratamento de escolha com maior recorrência. Assim sendo, cabe destacar que testes psicométricos anteriores realizados na PPSC verificaram que os tratamentos para controle do câncer, principalmente a quimioterapia, influenciaram negativamente na funcionalidade da criança e do adolescente com câncer^(22)^. Com isso, ressalta-se a necessidade de aplicação da escala em uma amostra consistente para novos testes psicométricos.

### Limitações do estudo

Este estudo apresenta algumas limitações importantes. A principal delas está relacionada ao fato de que a escala ainda não passou pelo processo de adaptação transcultural em outro país, o que dificulta a discussão e a comparação dos resultados com estudos internacionais. Além disso, é relevante destacar que a pesquisadora principal não participou da coleta de dados, o que limitou a realização de mais testes psicométricos. Outro aspecto a considerar é a redução das atividades para crianças e adolescentes no ambiente hospitalar devido à restrição da circulação de pessoas nas unidades, como medida de controle da disseminação do vírus SARS-CoV-2.

### Contribuições para a Área da Enfermagem e Saúde Pública

Este estudo contribui para o avanço do conhecimento científico e para a enfermagem com a disponibilidade de uma escala em português, adaptada para a realidade brasileira, que avalia a funcionalidade com base nas atividades e brincadeiras desenvolvidas pelas crianças e adolescentes, com o intuito de favorecer a autonomia e independência delas durante a hospitalização, promovendo a alta precoce para o domicílio. A escala PPSC, na versão em português, pode ser utilizada na avaliação de outras escalas de forma fidedigna, por ter sido traduzida e adaptada com cientificidade através de protocolos reconhecidos e amplamente utilizados pela comunidade científica. Desta forma, estudos de validação de instrumentos poderão utilizar a versão adaptada para validação concorrente e convergente acerca de distúrbios que causem diminuição da funcionalidade da criança e do adolescente ou alteração na qualidade de vida.

## CONCLUSÕES

A escala foi traduzida e adaptada para o português brasileiro e demonstrou ser relevante para a área de saúde e enfermagem por avaliar a funcionalidade das crianças e adolescentes com câncer, propiciando a identificação e estimulação das atividades e brincadeiras de acordo com a capacidade funcional individual, como também indica a realização de novos estudos que necessitam avaliar a funcionalidade, de forma fidedigna, com uso de uma escala de acordo com a realidade brasileira.
